# Recruitment strategies to promote uptake of cervical cancer screening in the West Region of Cameroon

**DOI:** 10.1186/s12889-022-12951-1

**Published:** 2022-03-19

**Authors:** Marie-Anne Pham, Khadidja Benkortbi, Bruno Kenfack, Eveline Tincho Foguem, Jessica Sormani, Ania Wisniak, Sophie Lemoupa Makajio, Engelbert Manga, Pierre Vassilakos, Patrick Petignat

**Affiliations:** 1 Faculty of Medicine, University of Geneva, Geneva, Switzerland; 2 Gynecology Division, Department of Pediatrics, Gynecology and Obstetrics, University Hospitals of Geneva, Geneva, Switzerland; 3 Faculty of Medicine and Pharmaceutical Sciences, University of Dschang, Dschang, Cameroon; 4 Department of Gynecology and Obstetrics, District Hospital of Dschang, Dschang, Cameroon; 5 Department of Gynecology and Obstetrics, Biyem-Assi District Hospital, Yaoundé, Cameroon; 6 School of Health Sciences, HES-SO University of Applied Sciences and Arts Western Switzerland, Geneva, Switzerland; 7 Population Epidemiology Unit, Department of Primary Care, University Hospitals of Geneva, Geneva, Switzerland; 8 Institute of Global Health, University of Geneva, Geneva, Switzerland; 9 Help and Reintegration Center for Disabled Youth, Obala, Cameroon; 10 Geneva Foundation for Medical Education and Research, Geneva, Switzerland

**Keywords:** Cervical cancer screening, Recruitment strategies, Community health workers, Cost-effectiveness

## Abstract

**Objectives:**

The World Health Organization’s (WHO) global strategy for cervical cancer elimination has set the target of 70% of women screened in all countries by 2030. Community sensitization through media is often used, but community health workers’ (CHW) involvement may contribute to improving screening coverage. We aimed to assess effectiveness and costs of two cervical cancer screening recruitment strategies conducted in a low-resource setting.

**Methods:**

The study was conducted in the West Region of Cameroon, in the Health District of Dschang, a community of 300,000 inhabitants. From September 2018 to February 2020, we recruited and screened women for cervical cancer in a single-visit prevention campaign at Dschang District Hospital. During the first 9 months, recruitment was only based on Community Information Channels (CIC) (e.g.. street banners). From the tenth month, participation of CHW was added in the community after training for cervical cancer prevention counselling. Population recruitment was compared between the two strategies by assessing the number of recruited women and direct costs (CHW costs included recruitment, teaching, certification, identification badge, flyers, transport, and incentives). The intervention’s cost-effectiveness was expressed using an incremental cost-effectiveness ratio (ICER).

**Results and discussion:**

During the period under study, 1940 women were recruited, HPV positive rate was 18.6% (*n* = 361) and 39 cases of cervical intraepithelial neoplasia grade 2 or worse (CIN2+) were diagnosed. Among included participants, 69.9% (*n* = 1356) of women were recruited through CIC as compared to 30.1% (*n* = 584) by CHW. The cost per screened woman and CIN2+ diagnosed was higher in the CHW group. The ICER was 6.45 USD or 16.612021Int’l$ per screened woman recruited by CHW. Recruitment in rural areas increased from 12.1 to 61.4% of all women included between CIC-led and CHW-led interventions. These outcomes highlight the importance of training, preparing, and deploying CHW to screen hard-to-reach women, considering that up to 45% of Cameroon’s population lives in rural areas.

**Conclusion:**

CHW offer an important complement to CIC for expanding coverage in a sub-Saharan African region such as the West Region of Cameroon. CHW play a central role in building awareness and motivation for cervical cancer screening in rural settings.

**Supplementary Information:**

The online version contains supplementary material available at 10.1186/s12889-022-12951-1.

## Background

Nearly 90% of cervical cancer (CC) deaths worldwide occur in low- and middle-income countries, with a mortality rate almost three times higher than in more economically developed countries [1]. Among countries with the highest CC burden, 19 of the top 20 are located in sub-Saharan Africa [2]. A key reason for persistent high morbidity and mortality is the lack of sufficient screening coverage [3]. The main challenges for introducing an efficient screening program in sub-Saharan countries are limited resources and health infrastructure, shortage of health care providers, a low level of awareness, and insufficient attention to women’s health, especially in rural populations [4, 5]. The result is that the vast majority of women do not have access to screening and treatment.

In response to this growing problem, the World Health Organization (WHO) has launched the 90–70-90 targets for 2030, aiming to eliminate CC [6]. These targets include (i) coverage of 90% of girls vaccinated, (ii) 70% of women screened, and (iii) treatment of 90% of women identified with cervical disease. To reach the second and third WHO targets, it is recommended to use high-performance HPV tests and associate screening with immediate treatment if needed (“screen-and-treat” approach) [7, 8].

The Health District of Dschang (West Region of Cameroon) has about 300,000 inhabitants. The Health District is divided into 22 health areas among which one is urban, 2 semi-urban and 19 rural [9]. In September 2018, we implemented a screening and treatment program in Dschang District Hospital, based on a single-visit approach called 3 T (Test, Triage and Treat). The ongoing program is scheduled over a five-year period (2018–2023) and follows the WHO’s recommendations to screen women between the ages 30–49 years at least once every 5 years. According to a national census, we estimated that about 18′000 women should be screened in the Health District of Dschang to reach the 70% coverage rate set by the WHO’s “90–70-90 targets” [6]. Therefore, an annual recruitment of 3′600 women was estimated to reach the second WHO target.

A program’s performance and its impact on CC prevention highly depends on the screening coverage achieved by reaching the targeted population. This may be enhanced by raising awareness through educational interventions. Outreach strategies to encourage participation in prevention programs may be viewed as low priority activities and may suffer from a lack of resources as they compete with other healthcare issues such as infectious diseases [10, 11]. Several challenges have been raised regarding the optimal recruitment strategies to inform women about screening and motivate them to participate. Screening interventions should also consider geographic conditions and the dispersion of the population living in these areas and be adapted to population needs.

Traditional choices for raising CC awareness in a large population within a short period of time are community information channels (CIC), such as advertising, radio, and television. However, the implication of community health workers’ (CHW) living in the community may contribute to improving education and motivation for screening, and therefore increase coverage. The definition of CHW varies according to different cultures and healthcare systems. In Cameroon, they are trusted community members, integrated into the community health system without any formal professional or paraprofessional medical training [12–17]. The purpose of this study was to analyze and compare the recruitment rates and costs of the two different recruitment strategies.

## Methods

### Setting

The Health District of Dschang is divided into 22 health areas, which we separated into four zones based on accessibility to the district hospital (e.g. distances, roads, weather, and transportation means available). Zone 1 was defined as the most accessible area (urban) and Zone 4 the least accessible (rural).

### CIC recruitment

From September 2018 to May 2019, recruitment was entirely based on announcements made and posters hung in women’s associations, churches, and integrated health centers (chief nurses of each center were informed of this project). In Dschang District Hospital’s waiting room, women were invited to participate in the screening program on a daily basis. Two large street banners were hung on the main tar road at the entrance and exit of Dschang for 1 month. Although the banners were hung in zone 1, this route is an access to district services and to markets where farmers weekly trade. Hence, it is used by inhabitants of all zones. The banners’ exposition time was restricted by local authorities as this location was regularly used to announce local events. Local radio announcements were broadcast twice a week for 1 month. The limited duration of broadcasting was due to budget constraints. Radio announcements were made in French which is the official language used in the district. We expected participants to spread information about this campaign to their relatives. The combination of these methods of recruitment is summarized as CIC.

### CHW recruitment

From June 2019 to February 2020, a recruitment strategy using CHW was added to the CIC intervention. At district level, district health managers were informed about the campaign and invited to participate by recruiting CHW. Selection was based on volunteer application without any prerequisites. CHW work in their village. They usually have a main job and act as CHW when called during public health activities. An incentive of 600 CFA (1 USD or 2.62021Int’l$) per woman recruited from June to September 2019 was given, which was then increased to 1000 CFA (1.68 USD or 4.32021Int’l$) since October 2019 to adequately cover cellphone and transportation fees.

In June 2019, 21 CHW attended a half-day informal training. These CHW were based in zone 1. In October 2019, 52 CHW from all health areas (zones 1 to 4) were enrolled in a two-day multi-modal training. Among the trained participants, 5 workers attended both sessions. The second session was based on the “WHO Toolkit for improving CWH Program and Service” [18, 19] and adapted to local barriers by regional caregivers. To differentiate CHW recruitment from CIC, CHW were given invitation vouchers to distribute to each woman they approached. CHW received their financial incentive according to the respective number of vouchers returned by participants attending screening.

### Screening process

Recruited participants were expected to be present at the screening unit at 9 am daily, where a one-hour health education session was provided by trained midwives. General information was given on sexually transmitted infections including HPV (its prevention, cancer development and treatment), contraceptive methods, intimate hygiene, and on the study project (inclusion, exclusion criteria, study procedures, planned follow-up). Participants were asked how they heard about the screening program and were encouraged to spread information about the campaign. Questions were addressed during this time. Participants filled a questionnaire on their socio-demographic characteristics and medical history and proceeded to vaginal self-sampling (Self-HPV) for primary screening. Samples were analyzed using the Xpert HPV assay® (GeneXpert®. Cepheid, 2015. Sunnyvale, California, USA). Results were available within 1 h. HPV-negative participants were advised to repeat screening in 5 years. HPV-positive women underwent triage by visual inspection after application of acetic acid (VIA) and Lugol’s iodine (VILI), and treatment with thermal ablation or large loop excision of the transitional zone (LLETZ) if required. Biopsy and endocervical curettage were performed on all HPV-positive patients for CC exclusion, quality control and further program evaluation. Further details can be found in previously published articles [20, 21].

### Data collection

Before completing their HPV test, participants filled a sociodemographic questionnaire distributed by midwives.

### Inclusion

We included for this analysis all women aged 30 to 49 years old living within Dschang’s Health District or its surroundings, who underwent an HPV test from September 2018 to February 2020. Exclusion criteria for HPV screening were pregnancy, hysterectomy, and vaginal bleeding. After verification of inclusion and exclusion criteria, volunteers provided informed written consent to participate in the study. To be counted in the CHW recruitment group, women had to present a CHW invitation voucher.

### Outcome measures

(i) A comparison of sociodemographic characteristics of women recruited by each method was performed with in-depth analysis for each zone of origin. (ii) The number of participants screened was assessed and costs for the implementation of CHW and CIC interventions compared and (iii) to assess the cost-effectiveness of CHW, the costs and screening recruitment outcomes associated with each intervention were compared to generate an incremental cost-effectiveness ratio (ICER). Costs of recruitment by CHW included workers’ recruitment, training supplies, certification, identification badges, vouchers, transportation, meals, accommodation, incentives, per diem, and miscellaneous materials. CIC costs included radio broadcasting, banners, and poster. Both groups included financial aid for women’s transportation to the screening center according to hospital accessibility from each health area. To highlight the actual field situation and its margin of error, we decided to compare the real-life cost-effectiveness (actual expenses, including incorrect patient transport financial aid), and the theoretical cost-effectiveness (expected expenses) generated by the CHW intervention to the cost-effectiveness of the CIC intervention. Costs are expressed in USD according to the exchange rate on March 1st, 2020 and, in international dollars to consider purchasing power parity.

### Statistical analysis

Quantitative data were stored and analyzed using Stata Statistical Software Release 16 (StataCorp LP, College Station, TX, USA). A descriptive analysis was conducted; categorical variables were summarized with frequencies and percentages, and continuous variables were summarized with means and standard deviations (SD). *P*-values were estimated using Pearson’s chi-squared test, Student’s t-test, and ANOVA test as appropriate. All analyses were 2-sided and *p*-values < 0.05 were considered statistically significant. Women’s socio-demographic and medical data were collected, stored, and managed by the secuTrial® online database. The calculated incremental cost-effectiveness ratio (ICER) was determined as the additional cost per screened woman by CHW, calculated as the difference between CHW costs and CIC costs divided by the difference of the number of screened women between CHWs and CIC.

### Ethical considerations

The study obtained approval from the Cantonal Ethics Board of Geneva, Switzerland (Commission cantonale d’éthique de la recherche [CCER], No. 2017–0110) and the Cameroonian National Ethics Committee for Human Health Research (No. 2018/07/1083/CE/CNERSH/SP).

## Results

### Population

**Fig. 1:**
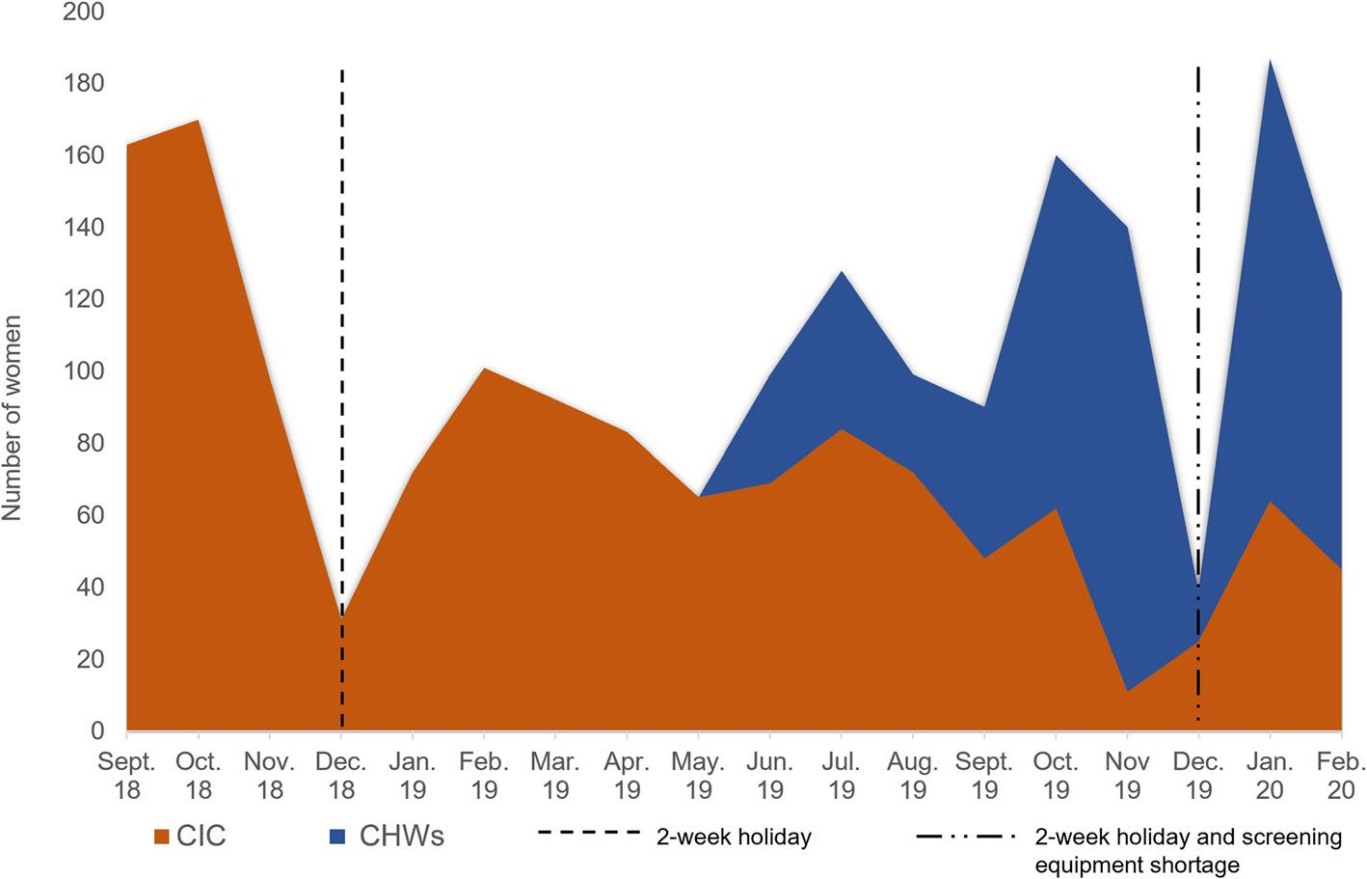
Recruitment progression from September 2018 to February 2020

**Fig. 2:**
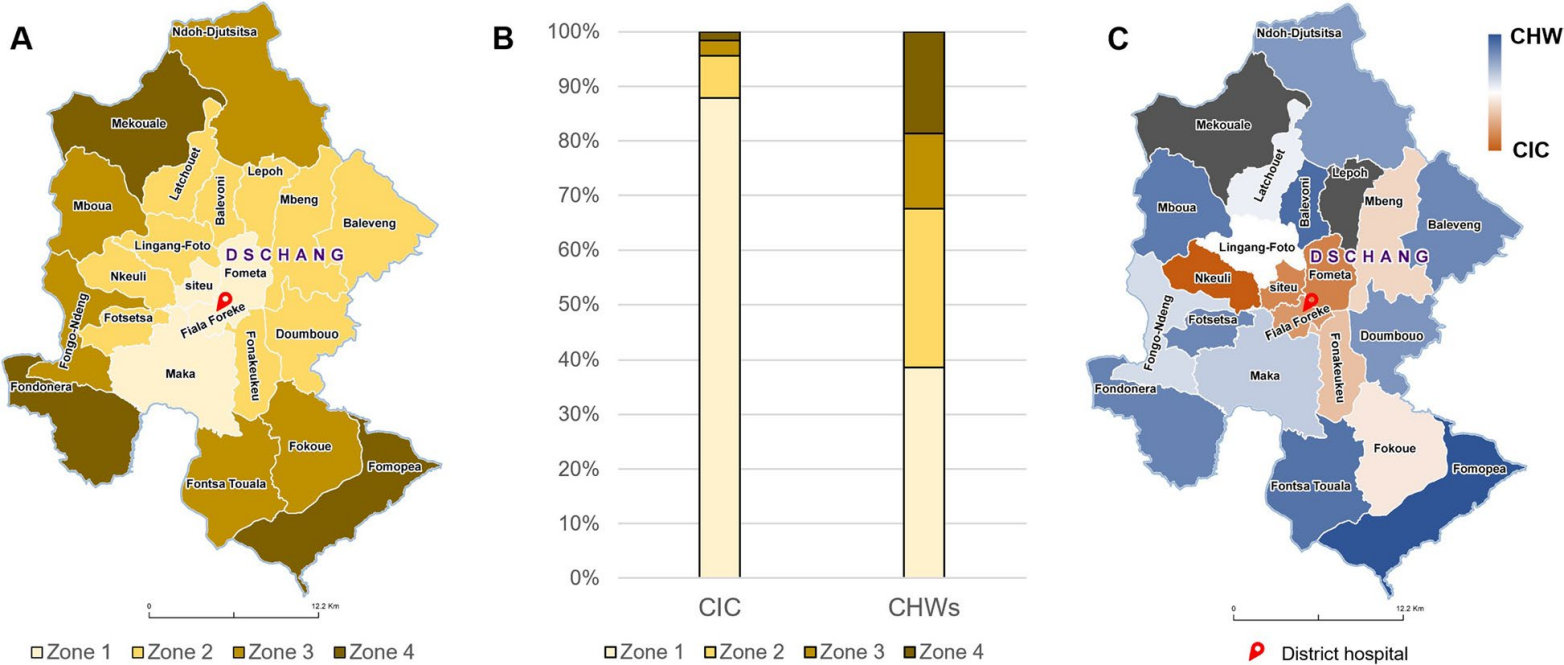
**A-C**. **A**. Dschang Health Area separated in 4 zones. Zone 1 being the most accessible areas (urban) and Zone 4 the least accessible areas (rural). **B**. Screening rate per zone according to recruitment method. **C**. Recruitment method predominance. Health areas in dark blue represent a predominance of women recruited by CHW, and orange represents health areas where recruitment was predominantly done through CIC. White color indicates that patients were equally recruited by CIC and CHW. In grey, two health areas were excluded from the analysis (Mekouale and Lepoh) as no CHW participated

A total of 1940 women were included during the study period, with an HPV positive rate of 18.6% (*n* = 361), and 39 CIN2+ (2.0%) lesions were diagnosed. In the CIC group, 1356 women (69.9%) were recruited and 28 CIN2+ (2.1%) lesions were detected. In the CHW group, 584 women (30.1%) were recruited and 11 CIN2+ (1.8%) lesions identified. Two hundred sixteen participants living outside the health district of Dschang were recruited in the CIC group, and 19 patients in the CHW group. Among the 68 CHW trained, eight did not recruit any participants. The recruitment progress is depicted in Fig. 1 showing reuptake of the recruitment trend when introducing CHW, and a sharp decrease in recruitment in December due to the annual closing of the Dschang Screening Unit for the winter holidays combined with equipment shortage during that period. Figure 2A-B shows the proportion of women recruited by district zone with each method. Using the CIC method, 87.89% of women were recruited in zone 1 (*n* = 1002), 7.72% in zone 2 (*n* = 88), 2.81% in zone 3 (*n* = 32) and 1.58% in zone 4 (*n* = 18). With the CHW method, 38.58% of women were recruited in zone 1 (*n* = 218); 29.03% in zone 2 (*n* = 164); 13.81% in zone 3 (*n* = 78); 18.58% in zone 4 (*n* = 105).

### CIC versus CHW recruitment

As shown in Table 1, the mean age of participants was 40.2 years old (SD ± 5.9). The two groups differed significantly on all socio-demographic variables (*p* = < 0.001). However, we did not observe any significant difference between both groups in the proportion of positive HPV test results, HIV self-reported status, and histology among HPV-positive women. Some variables had missing data as a few participants did not answer all questions. Populations recruited by CHW compared to CIC accounted for more divorced and widowed women (11% vs 5.8%) and fewer single women (4.3% vs 8.9%). The predominant education level in the CIC group was secondary (56.0%) and tertiary (23.25%), while in the CHW group, the predominant education level was primary and secondary with 46.9% in each sub-category. Employed and self-employed women were the most represented in the CIC-led intervention at 35.4 and 30.4%, respectively, whereas in the CHW-led recruitment, women were mostly farmers (42.6%) and housewives (25.2%). The unemployment rate in our sample was 0.4%. Women recruited by CHW often had more than 4 children (69.2%) compared to CIC-recruited women, among which 48,8% had between 1 and 4 children and 46.2% had more than 4 children. Tobacco consumption was higher among women in the CHW group (4.6% vs 0.5%). Most women (69.8%) did not use any form of contraceptive. Condom usage was reported by 13.9% in the CIC group compared to 3.6% in the CHW group. Intra-uterine devices, hormonal implants, and injections were used as a contraceptive method for 14.7% of participants in the CIC recruitment group and 20.0% of participants in the CHW group. Previous CC screening was reported by 24.3% in the CIC-led intervention and 3.9% in the CHW-led intervention.

**Table 1:** Baseline sociodemographic, reproductive health, and clinical characteristics according to CHW and CIC groups

	CIC, ***n*** (%)	CHW, ***n*** (%)	Total, ***n*** (%)	***P***-value*
**Variable**				
Participants recruited	1356 (69.9%)	584 (30.1%)	1940 (100%)	
Age (years), mean ± SD	39.4 (±5.9)	41.9 (±5.6)	40.2 (±5.9)	< 0.001
Marital status				< 0.001
Single	121 (8.9%)	25 (4.3%)	146 (7.5%)	
Married/In a relationship	1155 (85.2%)	494 (84.6%)	1649 (85%)	
Divorced/widowed	78 (5.8%)	64 (11.0%)	142 (7.3%)	
Education				< 0.001
Unschooled	5 (0.4%)	9 (1.5%)	14 (0.7%)	
Primary education	275 (20.3%)	274 (46.9%)	549 (28.3%)	
Secondary education	759 (56.0%)	274 (46.9%)	1033 (53.3%)	
Tertiary education	315 (23.2%)	24 (4.1%)	339 (17.5%)	
Employment status				< 0.001
Employed	480 (35.4%)	62 (10.6%)	542 (27.9%)	
Self-employed	412 (30.4%)	121 (20.7%)	533 (27.5%)	
Farmer	130 (10.0%)	249 (42.6%)	379 (19.5%)	
Housewife	274 (20.2%)	147 (25.2%)	421 (21.7%)	
Student	50 (3.7%)	4 (0.7%)	54 (2.8%)	
Unemployed	8 (0.6%)	0	8 (0.4%)	
Age at menarche (years), mean ± SD	14.6 (1.8)	14.9 (1.7)	14.7 (1.8)	< 0.001
Age at first intercourse, mean ± SD	18.0 (2.9)	17.48 (2.4)	17.9 (2.8)	< 0.001
Number of sexual partners, median (IQR)	3 (2–5)	3 (2–4)	3 (2–5)	
Age at first delivery (years), mean ± SD	21.3 (5.8)	19.8 (4.3)	20.9 (5.4)	< 0.001
Parity				< 0.001
Nulliparous	65 (4.8%)	13 (2.2%)	78 (4.0%)	
1–4	662 (48.8%)	166 (28.4%)	828 (42.9%)	
> 4	627 (46.2%)	404 (69.2%)	1031 (53.1%)	
Tabaco consumption				< 0.001
Yes	7 (0.5%)	27 (4.6%)	34 (1.8%)	
None	1347 (99.3%)	555 (95.0%)	1902 (98.0%)	
Contraception				< 0.001
None	924 (68.1%)	430 (73.6%)	1354 (69.8%)	
Condom	189 (13.9%)	21 (3.6%)	210 (10.8%)	
Hormonal pill	23 (1.7%)	8 (1.4%)	31 (1.6%)	
DIU/ implant/ injection	199 (14.7%)	117 (20.0%)	316 (16.3%)	
Other	16 (1.2%)	5 (0.9%)	21 (1.1%)	
Previous cervical cancer screening				< 0.001
None	1025 (75.6%)	560 (95.9%)	1585 (81.7%)	
Yes	329 (24.3%)	23 (3.9%)	352 (18.1%)	
HIV status (self-reported)				0.791
Negative	1327 (97.9%)	574 (98.3%)	1901 (98.0%)	
Positive	27 (2.0%)	9 (1.5%)	36 (1.9%)	
HPV testing results				0.287
Negative	1096 (80.8%)	484 (82.9%)	1580 (81.4%)	
Positive	260 (19.2%)	100 (17.1%)	360 (18.6%)	
HPV-16/18/45	59 (4.4%)	24 (4.1%)	83 (4.3%)	
Other HPV	215(15.9%)	86 (14.7%)	301 (15.5%)	
Histology (% of HPV positive women)				0.437
Normal	179 (68.8%)	62 (62%)	241 (66.9%)	
CIN1	44 (16.9%)	18 (18%)	62 (17.2%)	
CIN2+	28 (10.8%)	11 (11%)	39 (10.8%)	

*Abbreviations*: *CHW* Community Health Workers, *CIC* Community Information Channels, *SD* Standard Deviation, *IQR* Interquartile range, *HIV* Human immunodeficiency virus, *HPV* Human papillomavirus, *n* number

*p-values were estimated using chi-squared test, t-test as appropriate

### Recruitment breakdown by zone

Socio-demographic differences between women recruited by CIC and CHW in the four zones are described in Table 2**.** Participants’ mean age varied between urban and rural areas, with women in zone 1 tending to be younger than those in zone 4 (*p* <  0.001). In zone 1, primary education only was attended by 17.4% of women in the CIC group contrasting with 39.5% of women recruited in the CHW group. Furthermore, secondary level and higher was reached by more participants in the CIC group than in the CHW-led intervention except for zone 4, (38.9% in the CIC and 49.5% in the CHW group). Tertiary education was attended by 25.6% in zone 1 recruited by CIC compared to only 7.8% in zone 1 within the CHW group. In the CIC-led intervention, women in zone 1 were more frequently employed (38.1%) and self-employed (33.1%), whereas in zone 2–4, women worked more frequently as farmers (34.1, 34.4 and 50% respectively). In the CHW-led intervention, most participants were self-employed in zone 1 (34.9%), and farmers in zones 2 (49.4%), 3 (92.3%) and 4 (76.2%). We also found that most unemployed women lived in zone 1 and were recruited through CIC. Women coming from zone 1 and recruited through CIC had fewer children than in other zones. Indeed, 49.7% women in zone 1 had between 1 and 4 children and 45.51% had more than 4 children, while in other subgroups, between 62.5 and 80.95% of participants had more than 4 children. Within the CIC-recruited group, most women who used condoms were in zone 1 (15.2%,) and zone 2 (13.6%). Participants who smoked the most were recruited by CHW and live in zone 2 (7.9%) and in zone 3 (12.8%). Variance in previous CC screening was also shown between women living in urban zones compared to those in rural zones and depending on the recruitment method. Among women recruited through CIC, 25.45% of those living in zone 1 had a history of previous CC screening, 15.9% in zone 2, 9.4% in zone 3 and 5.6% in zone 4 (*p* = 0.092). Rates of previous screening were generally lower in women recruited by CHW, where 7.8% of women in zone 1 had a previous CC screening, 1.2% in zone 2, 2.6% in zone 3 and 1.9% in zone 4 (*p* = 0.017).

**Table 2:** Baseline sociodemographic, reproductive health, and clinical characteristics according to CHW and CIC groups and Zone subgroups

Variable	CIC (1140/1356), ***n*** (%)				***P***-Value	CHW (565/584), n (%)				***P***- Value
	Zone 1	Zone 2	Zone 3	Zone 4		Zone 1	Zone 2	Zone 3	Zone 4	
Participants recruited n (%)	1002 (87.9%)	88 (7.7%)	32 (2.8%)	18 (1.6%)		218 (38.6%)	164 (29.0%)	78 (13.8%)	105 (18.6%)	
Age (years), mean ± SD	39.1 (±5.9)	41.4 (±5.6)	41.5 (±5.4)	42.3 (±4.3)	< 0.001	40.9 (±5.7)	43.1 (±5.4)	43 (±5.5)	41.9 (±5.3)	< 0.001
Marital status					< 0.001					0.023
Single	81 (8.1%)	3 (3.4%)	3 (9.4%)	0		15 (6.9%)	6 (3.7%)	2 (2.6%)	0	
Married/In a relationship	870 (86.8%)	76 (86.4%)	25 (78.1%)	13 (72.2%)		189 (86.7%)	133 (81.1%)	65 (83.3%)	91 (86.7%)	
Divorced/widowed	50 (5.0%)	9 (10.2%)	4 (12.5%)	5 (27.8%)		14 (6.4%)	25 (15.2%)	11 (14.1%)	13 (12.4%)	
Education					< 0.001					< 0.001
Unschooled	3 (0.3%)	2 (2.3%)	0	0		1 (0.5%)	6 (3.7%)	1 (1.3%)	1 (1.0%)	
Primary education	174 (17.4%)	25 (28.4%)	12 (37.5%)	11 (61.1%)		86 (39.5%)	85 (51.8%)	47 (60.3%)	51 (48.6%)	
Secondary education and higher	824 (82.2%)	61 (69.3%)	20 (62.5%)	7 (38.9%)		131 (60.1%)	72 (43.9%)	30 (38.5%)	52 (49.5%)	
Employment status					< 0.001					< 0.001
Employed	382 (38.1%)	16 (18.2%)	8 (25%)	3 (16.7%)		36 (16.5%)	11 (6.7%)	10 (12.8%)	3 (2.9%)	
Self-employed	332 (33.1%)	14 (15.9%)	2 (6.3%)	2 (11.1%)		76 (34.9%)	30 (18.3%)	6 (7.7%)	6 (5.7%)	
Farmer	55 (5.5%)	30 (34.1%)	11 (34.4%)	9 (50%)		33 (15.1%)	81 (49.4%)	50 (92.3%)	80 (76.2%)	
Unemployed, Housewife and student	232 (23.2%)	28 (31.4%)	11 (34.4%)	4 (22.2%)		73 (33.5%)	42 (25.6%)	12 (15.4%)	15 (14.3%)	
Age at menarche (years), mean ± SD	14.6 (1.8)	14.6 (1.8)	15.1 (1.8)	14.7 (1.6)	0.389	15.0 (1.7)	14.7 (1.6)	14.9 (1.7)	14.9 (1.8)	0.583
Age at first intercourse, mean ± SD	18.1 (2.9)	17.3 (1.9)	17.9 (2.8)	16.3 (1.5)	0.004	17.9 (2.5)	17.2 (2.0)	17.4 (2.4)	17.0 (2.5)	0.012
Number of sexual partners, median (IQR)	3 (2–5)	3 (2–5)	3 (2–4)	3 (2.8–4.3)		3 (2–4)	3 (2–4)	2 (1–4)	3 (2–5)	
Age at first delivery (years), mean ± SD	21.5 (5.9)	19.6 (4.2)	20.6 (4.7)	19.7 (2.7)	0.013	19.9 (4.5)	19.59 (3.7)	20.2 (4.1)	19.3 (5.0)	0.777
Parity					0.001					0.001
Nulliparous	47 (4.7%)	3 (3.4%)	1 (3.1%)	0		5 (2.3%)	3 (1.8%)	1 (1.3%)	2 (1.9%)	
1–4	498 (49.7%)	27 (30.7%)	11 (34.4%)	4 (22.2%)		72 (33.0%)	46 (28.1%)	19 (24.4%)	17 (16.2%)	
> 4	456 (45.5%)	58 (65.9%)	20 (62.5%)	14 (77.8%)		141 (64.7%)	115 (70.1%)	58 (74.4%)	85 (81.0%)	
Contraception					0.002					0.120
None	659 (65.8%)	61 (69.3%)	28 (87.5%)	14 (77.8%)		160 (73.4%)	131 (79.9%)	57 (73.1%)	66 (62.9%)	
Condom	152 (15.2%)	12 (13.6%)	2 (6.3%)	1 (5.6%)		8 (3.7%)	6 (3.7%)	3 (3.9%)	3 (2.9%)	
Other	190 (19.0%)	15 (17.1%)	2 (6.3%)	3 (16.7%)		50 (22.9%)	26 (15.9%)	18 (23.1%)	34 (32.4%)	
Previous cervical cancer screening					0.092					0.017
None	746 (74.5%)	74 (84.1%)	29 (90.6%)	17 (94.4%)		201 (92.2%)	162 (98.8%)	76 (97.4%)	102 (97.1%)	
Yes	255 (25.5%)	14 (15.9%)	3 (9.4%)	1 (5.6%)		17 (7.8%)	2 (1.2%)	2 (2.6%)	2 (1.9%)	
HIV status (self-reported)					0.004					0.596
Negative	949 (94.7%)	82 (93.2%)	26 (81.3%)	17 (94.4%)		213 (97.7%)	159 (97.0%)	75 (96.2%)	100 (95.2%)	
Positive	39 (3.9%)	5 (5.7%)	3 (9.4%)	0		3 (1.4%)	3 (1.8%)	2 (2.6%)	1 (1.0%)	
HPV testing results					0.091					0.741
Negative	817 (81.54%)	76 (86.36%)	27 (84.38%)	12 (66.67%)		180 (82.57%)	135 (82.32%)	64 (82.05%)	87 (82.86%)	
Positive	185 (18.46%)	12 (13.64%)	5 (15.63%)	6 (33.33%)		38 (17.43%)	29 (17.68%)	14 (17.95%)	18 (17.14%)	

*Abbreviations*: *CHW* Community Health Workers, *CIC* Community Information Channels, *SD* Standard Deviation, *IQR* Interquartile range, *HIV* Human immunodeficiency virus, *HPV* Human papillomavirus, *n* Number

### Screening rate

Figure 2C shows recruitment method predominance by health area. We observed a predominance for CHW recruitment in areas distant from the district center.

The cost-effectiveness analysis of recruitment is presented in Table 3.

**Table 3:** Cost-analysis of recruitment. The incremental cost-effectiveness ratio (ICER) is determined as the additional cost per screened woman calculated as the difference between CHW costs and CIC costs divided by the additional of number of screened women due to CHW

Variable	CIC (USD)	CHW (USD)
Recruited patients (n)	1356	584
Patients’ transport reimbursement	1345.74	1845.87
Street Banners (*n* = 2)	184.92	N/A
Radiobroadcast (*n* = 4)	33.62	N/A
Flyers (*n* = 1000)	42.03	N/A
CHW ‘s training	N/A	2657.86
CHW wages	N/A	870.40
**Total costs**	1606.31	5374.13 (training included) 2716.27 (training excluded)
**Costs per recruited woman**		
ACER	1.18	9.20 (training included) 4.65 (training excluded)
Incremental additional cost	N/A	3767.82 (training included) 1109.06 (training excluded)
ICER in USD	N/A	6.45 (training included) 1.90 (training excluded)
ICER in 2021Int’l$*	N/A	16.61 (training included) 4.89 (training excluded)

*Abbreviations*: *CHW* Community Health Workers, *CIC* Community Information Channels, *ICER* Incremental cost-effectiveness ratios, *ACER* Average cost effectiveness ratio, *n* Number, *N/A* Not applicable

### Cost analysis

A detailed breakdown of CHW training costs for each session is presented in the supplemental table. The June session cost a total of 694.98 USD (cost per trained CHW was 33.09 USD for 21 CHW). The October session’s total cost was 1962.88 USD (cost per trained CHW was 37.75 USD for 52CHW). CIC costs include 33.62 USD for four radio broadcasts, 184.92 USD for two street banners, 42.03 USD for a thousand poster. Based on the onsite accounting book, the patients’ transport cost was 1411.30 USD without distinction between the two intervention groups. To compare the two groups, the theoretical patients’ transport cost is presented on Table 3. This was calculated based on the predefined amount allocated to each participant according to hospital accessibility from each health area and amounted to 1845.87 USD for the CHW-led intervention and 1345.74 USD for the CIC-led intervention. The financial incentives paid to CHW were calculated to be around 1141.57 USD (based on receipts when available, and theoretically calculated for participants with missing receipts). The theoretical cost of CHW incentive, based on the total number of women recruited through CHW was 870.40 USD. The average cost per CIC-recruited woman was 1.18 USD compared to 9.20 USD per CHW-recruited woman. Based on theoretical costs, the ICER was 6.45 USD or 16.612021Int’l$ per screened woman recruited by CHW. The average cost per CIN2+ lesion diagnosed was 57.37 USD in the CIC group compared to 488.56 USD in the CHW group.

## Discussion

The global WHO strategy for cervical cancer elimination recommends that each country should meet the 90–70-90 targets by 2030 [6]. Achieving and sustaining the second target (70% of participation rate to screening with a high-performance test) will be one of the most challenging issues for many LMICs. For example, in Cameroon, it was estimated that the cervical cancer screening participation rate in a woman’s lifetime is less than 10% [22]. This condition is one of the main reasons for the high cervical cancer incidence and mortality among middle-aged women in the country [8]. Our aim was to explore the effectiveness and costs of two different recruitment strategies in encouraging women to have a screening test.

Media-based information for public education about health-related issues are frequently used in national campaigns in Cameroon [23–25]. However, according to the 2018 Demographic and Health Survey in Cameroon, within the West Region, 38.1% of women were not exposed to any television, radio, or newspapers, 56.5% of women watched television and 22.4% listened to radio at least once a week [22]. This aspect is crucial for any decision making related to information spreading. Considering this data, radio broadcasting in our context may not be the most efficient strategy compared to television-based interventions. However, the latter may be more expensive. Data is still limited about the impact of encouraging behavior changes related to health services, and the resulting cost per person screened.

Efficiency results for screening coverage must consider that CIC and oral communication within the community co-existed with CHW-led interventions during the second period under study. Several women recruited by CHW could have been screened without mentioning the CHW referral, which would lead to their misclassification. Community-spread communication co-existed with the CHW-led intervention and probably also increased recruitment in each group; thus, CHW’s impact could be greater than we assumed. At the screening center, warm welcome can lead to a positive experience and favor recruitment.

CHW-led interventions constitute an important step to increase participation in cervical cancer screening programs. They contribute to optimizing the participation as they use their cultural knowledge and ensure that message are delivered in a culturally appropriate fashion according to women’s preferences and needs in rural areas where the screening rate is very low, which differ from those of women living closer to the city [14, 26]. As shown in Tables 1 and 2, women recruited by CHW tended to be less educated, had more children, used fewer condoms, and consumed more tobacco. Participant knowledge about cervical cancer in rural areas may not be the same as women living closer to the hospital. Studies have suggested that higher cervical cancer awareness is found among women living in an urban environment due to internet and media access [27]. It has been established that a lack of information and awareness about screening centers’ location, the cost of screening, available time, and geographical conditions are the main barriers to CC screening [28–31].

In our study, CIC were used to convey an invitation to get screened. However, in other studies, media used as an educational tool appeared as effective as CHW interventions to raise awareness about the importance of CC screening, although lay health workers were more effective to change screening behaviors through encouragement and logistical support [32, 33].

In our study, CIC appeared to be most suitable for women living close to the city center, while CHW improved recruitment coverage in rural areas. CHW not only enhanced recruitment outside urban areas, but they were also able to engage with and invite more women from a different socio-demographic population to be screened, including in zone 1. To avoid a bottleneck effect due to limited capacities at the screening center, one strategy could be to start by using CIC, before gradually implementing CHW intervention. A probable reason for a higher history of previous cervical cancer screening among participants from zone 1 in the CIC group was an increase of awareness and a built trust throughout a previous screening campaign led in Dschang in 2015, in addition to the 20 years of collaboration with our research team in Cameroon [21].

Transportation and childcare have been previously reported as screening barriers [26]. Our screening recruitment heavily depended on rainy seasons as roads were impassible. Moreover, financial transport aid was an essential aspect of our strategy as women living in rural areas had to travel for many hours. The CHW-led intervention helped to decrease these barriers as they recruited hard-to-reach women with multiple children and informed them about the financial subsidies for transportation.

The cost per screened woman and CIN2+ diagnosed was higher in the CHW group. While the media campaign was most efficient in zone 1, the higher recruitment of women in rural areas by CHW highlights the importance of training, preparing, and deploying CHWs to screen hard-to-reach women, especially considering that almost 45% of the Cameroonian population lives in a rural area [34]. Undetected cervical lesions potentially leading to cervical cancer also increase overall costs not only for the healthcare system but can cause direct and indirect costs for affected women and their families, such as cancer management costs, or loss of income due to disease, disability or death.

When possible, CHW selection should be based on abilities and long term motivation, and their work should be adequately compensated to avoid having inactive workers that need to be replaced by newly trained personnel, which would increase the screening cost [13, 35]. Training in October 2019 was more expensive in total than the first session in June 2019; however, the investment was similar if we consider the expense per CHW trained. Improving CHW knowledge is recognized as a key factor to a successful recruitment intervention [16]. This was evidenced during the October session based on a multi-modal training, which was followed by an increase of screened women.

Strategies with multiple visits to get screened, treated, and followed up may decrease screening effectiveness and increase the overall cost of cancer prevention per woman due to loss to follow-up [12, 36]. In our setting, the 3 T strategy led only to a 1.1% loss to follow-up and has the potential to increase program effectiveness as barriers for Cameroonian women include “low health literacy, poverty, lack of resources, and geographical conditions” [20]. However, additional follow-up visits after treatment may increase the need for CHW, as studies have shown that in-person follow-up could be a cost-effective approach to keep women in the screening process [12]. In this study, we only focused on the cost of screening recruitment; however, further studies are needed to assess the full financial and social burden through a cost-benefit analysis of an HPV “screen and treat” program in Dschang. In sub-Saharan Africa, most women dying of cervical cancer are around 50 years old, and DALYs caused by CC were estimated ats 641 years per 100′000 women [21, 37].

The large sample size and heterogeneity of the population regarding social and demographic characteristics are the major strengths of this study. Real-world conditions and thus the amount paid for equipment, supplies, and labor did not reflect theoretical costs. Health area attribution discordances and village overlap between two health areas/zones could have led to misclassification, as well as inexact cost and recruitment rate estimates, in addition to some miscommunication that led to incorrect patient reimbursement cost. Moreover, measuring the success rate of the CHW-led intervention could have allowed a more detailed analysis of the cost-effectiveness of CHW service. Indeed, the ratio of CHW-approached to screened women is currently unknown. Since recruitment strategies were not led simultaneously, a uniquely CHW-led intervention might have enrolled less participants as some women had already been informed through CIC. Furthermore, as various strategies (e.g. radio broadcasting, street banners) within the CIC intervention were led discontinuously throughout the study period, it is difficult to establish the effectiveness of each individually. Another limitation is that some women recruited by CHW might have eventually attended screening without CHW intervention.

## Conclusion

Combining both CIC and CHW approaches according to regional context appeared as the most efficient strategy for increasing recruitment among the target population. CHW play a central role in building awareness and motivation for cervical cancer screening among rural populations. Further studies are needed to explore innovative community-based interventions as effective ways to improve recruitment of the target population.

## Supplementary Information

**Additional file 1: Supplemental Table.** Detailed breakdown of CHWs training costs for each session.

## Data Availability

Data are available upon reasonable request. In accordance with the journal’s guidelines, we will provide our data for the reproducibility of this study in other centers if such is requested.

## Abbreviations

WHO: World Health Organization’s
HPV: Human papillomavirus
CHW: Community Health Workers
CIC: Community Information Channels
ICER: Incremental Cost-Effectiveness Ratio
CC: Cervical Cancer
SD: Standard Deviation
IQR: Interquartile range
HIV: Human immunodeficiency virus
N: Number

## References

[1] International Agency for Research on Cancer, World Health Organization. Global Cancer Observatory. Estimated cancer incidence, mortality and prevalence worldwide in 2018: cervical cancer. Available from: https://gco.iarc.fr/today/data/factsheets/cancers/23-Cervix-uteri-fact-sheet.pdf. [cited 2021 Feb 17]

[2] World Health Organization Regional Office for Africa. Infographics Cervical Cancer. Available from: https://www.afro.who.int/sites/default/files/health_topics_infographics/WHO_INFOgraphics_CervicalCancer.pdf. [cited 2021 Feb 18]

[3] International Agency for Research on Cancer. IARC handbooks of cancer prevention: volume 10—cervix cancer screening. 2005. Available from: https://publications.iarc.fr/Book-And-Report-Series/Iarc-Handbooks-Of-Cancer-Prevention/Cervix-Cancer-Screening-2005 [cited 2021 Feb 17]

[4] Denny L, Anorlu R (2012). Cervical cancer in Africa. Cancer Epidemiol Biomark Prev Publ Am Assoc Cancer Res Cosponsored Am Soc Prev Oncol.

[5] Ochomo EO, Atieli H, Gumo S, Ouma C (2017). Assessment of community health volunteers’ knowledge on cervical cancer in Kadibo division, Kisumu County: a cross sectional survey. BMC Health Serv Res.

[6] World Health Organization. Global strategy to accelerate the elimination of cervical cancer as a public health problem. 2020. Available from: https://apps.who.int/iris/bitstream/handle/10665/336583/9789240014107-eng.pdf [cited 2021 Feb 17]

[7] World Health Organization (2013). WHO guidelines for screening and treatment of precancerous lesions for cervical cancer prevention.

[8] World Health Organization, World Health Organization, Reproductive Health and Research. Comprehensive cervical cancer control: a guide to essential practice 2014. Available from: http://apps.who.int/iris/bitstream/10665/144785/1/9789241548953_eng.pdf?ua=1 [cited 2021 Mar 1] 25642554

[9] Bureau central des recensements et des études de population du Cameroun (BUCREP) (2010). Troisième recensement général de la population et de l’habitat (3e RGPH, 2005).

[10] Busolo D, Woodgate R. Cancer prevention in Africa: a review of the literature. Glob Health Promot. 2014;22(2):31–9. 10.1177/1757975914537094 25027971

[11] Anaman-Torgbor J, Angmorterh SK, Dordunoo D, Ofori EK. Cervical cancer screening behaviours and challenges: a sub-Saharan Africa perspective. Pan Afr Med J 2020;36(97). 10.11604/pamj.2020.36.97.19071 PMC7392861 32774656

[12] Mezei AK, Armstrong HL, Pedersen HN, Campos NG, Mitchell SM, Sekikubo M (2017). Cost-effectiveness of cervical cancer screening methods in low- and middle-income countries: a systematic review. Int J Cancer.

[13] O’Donovan J, O’Donovan C, Nagraj S (2019). The role of community health workers in cervical cancer screening in low-income and middle-income countries: a systematic scoping review of the literature. BMJ Glob Health.

[14] Wong CL, So WKW, Chan DNS, Choi KC, Rana T (2019). A community health worker-led multimedia intervention to increase cervical cancer screening uptake among south Asian women: study protocol for a cluster randomized wait-list controlled trial. Trials..

[15] Vaughan K, Kok MC, Witter S, Dieleman M. Costs and cost-effectiveness of community health workers: evidence from a literature review. Hum Resour Health. 2015;13(71). 10.1186/s12960-015-0070-y PMC4557864 26329455

[16] Kienen N, Bittencourt L, Pelloso SM, Consolaro MEL, Castle PE, Partridge EE (2018). Cervical Cancer Screening among Underscreened and Unscreened Brazilian Women: Training Community Health Workers to be Agents of Change. Prog Community Health Partnersh.

[17] Lewin S, Munabi-Babigumira S, Glenton C, Daniels K, Bosch-Capblanch X, van Wyk BE (2010). Lay health workers in primary and community health care for maternal and child health and the management of infectious diseases. Cochrane Database Syst Rev.

[18] Crigler L, Hill K, Furth R, Bjerregaard D. Community Health Worker Assessment and Improvement Matrix (CHW AIM): A Toolkit for Improving CHW Programs and Services. USAID Health Care Improvement Project. Bethesda: University Research Co., LLC (URC); 2013. Available from: https://www.who.int/workforcealliance/knowledge/toolkit/CHWAIMToolkit_Revision_Sept13.pdf [cited 2021 Mar 1]

[19] World Health Organization. Cervical cancer screening and management of cervical pre-cancers: training of community health workers. World Health Organization. Regional Office for South-East Asia; New Delhi, India. 2017. Available from: https://apps.who.int/iris/handle/10665/279798 [cited 2021 Mar 1]

[20] Kunckler M, Schumacher F, Kenfack B, Catarino R, Viviano M, Tincho E (2017). Cervical cancer screening in a low-resource setting: a pilot study on an HPV-based screen-and-treat approach. Cancer Med.

[21] Vassilakos P, Tebeu P-M, Halle-Ekane G, Sando Z, Kenfack B, Baumann F, et al. Vingt années de lutte contre le cancer du col utérin en Afrique subsaharienne - Collaboration médicale entre Genève et Yaoundé. Rev Med Suisse. 2019;5(642):601–5. 30865394

[22] Institut National de la Statistique (INS), et ICF. Enquête Démographique et de Santé du Cameroun 2018. Ministère de la santé publique du Cameroun. Yaoundé, Cameroun et Rockville, Maryland, USA, 2020. Available from: https://www.minsante.cm/site/?q=fr/content/enqu%C3%AAte-d%C3%A9mographique-de-sant%C3%A9-v2018. [cited 2021 Apr 11].

[23] Dzudie A, Djomou A, Ba H, Njume E, Ndom MS, Mfekeu LK (2019). MMM17-Cameroon, analysis and opportunities—sub-Saharan Africa. Eur Heart J Suppl J Eur Soc Cardiol.

[24] Bowen HL (2013). Impact of a mass media campaign on bed net use in Cameroon. Malar J.

[25] Babalola S, Figueroa ME, Krenn S (2017). Association of Mass Media Communication with contraceptive use in sub-Saharan Africa: a Meta-analysis of demographic and health surveys. J Health Commun.

[26] Elliott PF, Belinson SE, Ottolenghi E, Smyth K, Belinson JL (2013). Community health workers, social support and cervical cancer screening among high-risk groups in rural Mexico. J Health Care Poor Underserved.

[27] Nkfusai NC, Cumber SN, Anchang KJ, Nji KE, Shirinde J, Nota DA. Assessment of the current state of knowledge and risk factors of cervical cancer among women in the Buea Health District, Cameroon. Pan Afr Med J. 2019;33(38) 10.11604/pamj.2019.33.38.16767 PMC6661163 31384353

[28] Eze JN, Umeora OU, Obuna JA, Egwuatu VE, Ejikeme BN (2012). Cervical cancer awareness and cervical screening uptake at the mater Misericordiae hospital, Afikpo, Southeast Nigeria. Ann Afr Med.

[29] Abotchie PN, Shokar NK (2009). Cervical cancer screening among college students in Ghana: knowledge and health beliefs. Int J Gynecol Cancer Off J Int Gynecol Cancer Soc.

[30] Lim JNW, Ojo AA. Barriers to utilisation of cervical cancer screening in sub Sahara Africa: a systematic review. Eur J Cancer Care (Engl). 2017;26(1). 10.1111/ecc.12444 26853214

[31] Donatus L, Nina FK, Sama DJ, Nkfusai CN, Bede F, Shirinde J, et al. Assessing the uptake of cervical cancer screening among women aged 25-65 years in Kumbo west Health District, Cameroon. Pan Afr Med J. 2019;33(106). 10.11604/pamj.2019.33.106.16975 PMC6713511 31489084

[32] Lam TK, McPhee SJ, Mock J, Wong C, Doan HT, Nguyen T (2003). Encouraging Vietnamese-American women to obtain pap tests through lay health worker outreach and media education. J Gen Intern Med.

[33] Mock J, McPhee SJ, Nguyen T, Wong C, Doan H, Lai KQ (2007). Effective lay health worker outreach and media-based education for promoting cervical Cancer screening among Vietnamese American women. Am J Public Health.

[34] Rural population (% of total population) - Cameroon | Data. Available from: https://data.worldbank.org/indicator/SP.RUR.TOTL.ZS?locations=CM&view=map. [cited 2021 Feb 15]

[35] Colón-López V, González D, Vélez C, Fernández-Espada N, Soler AF, Escobar KA (2017). Community-academic partnership to implement a breast and cervical Cancer screening education program in Puerto Rico. P R Health Sci J.

[36] Goldhaber-Fiebert JD, Denny LE, De Souza M, Wright TC, Kuhn L, Goldie SJ (2005). The costs of reducing loss to follow-up in south African cervical cancer screening. Cost Eff Resour Alloc.

[37] Soerjomataram I, Lortet-Tieulent J, Parkin DM, Ferlay J, Mathers C, Forman D (2012). Global burden of cancer in 2008: a systematic analysis of disability-adjusted life-years in 12 world regions. Lancet.

