# Supplementary material for: Recruitment strategies to promote uptake of cervical cancer screening in the West Region of Cameroon
Source: BMC Public Health. 2022 Mar 19;22:548. doi: 10.1186/s12889-022-12951-1 (PMC8933918; doi:10.1186/s12889-022-12951-1)
Supplement: Supplementary file 1 — Additional file 1: Supplemental Table. Detailed breakdown of CHWs training costs for each session. [file 12889_2022_12951_MOESM1_ESM.docx]

**Supplemental Table: detailed breakdown of CHWs training costs for each session**

|  | June session (1-day session for 21 CHW) in USD | October session (2-day sessions for 52 CHW) in USD |
| --- | --- | --- |
| Recruitment fees | 462.31 |  |
| Trainer salary | 134.49 | 268.98 |
| Printing and supplies fees | 14.12 |  |
| Training supplies and miscellaneous materials |  | 100.87 |
| CHW's mentoring/coaching fees | 84.06 |  |
| Certification and identification badges |  | 44.89 |
| Transportation |  | 200.89 |
| Meals |  | 481.47 |
| Accommodation |  | 84.06 |
| Per diem |  | 781.72 |
| **Total** | **694.98** | **1962.88** |
| **Cost per CHW trained** | **33.09** | **37.75** |
